# Comparison of Physicochemical and Structural Properties of Acid-Soluble and Pepsin-Soluble Collagens from Blacktip Reef Shark Skin

**DOI:** 10.3390/md20060376

**Published:** 2022-06-02

**Authors:** Baolin Ge, Chunyu Hou, Bin Bao, Zhilin Pan, José Eduardo Maté Sánchez de Val, Jeevithan Elango, Wenhui Wu

**Affiliations:** 1 Department of Marine Pharmacology, College of Food Science and Technology, Shanghai Ocean University, Shanghai 201306, China; 13561936983@163.com (B.G.); ytxiaoyu@163.com (C.H.); bbao@shou.edu.cn (B.B.); zlpan4869@163.com (Z.P.); 2 Department of Biomaterials Engineering, Faculty of Health Sciences, UCAM-Universidad Católica San Antonio de Murcia, Guadalupe, 30107 Murcia, Spain; jemate@ucam.edu

**Keywords:** blacktip skin collagens, amino acid profile, protein pattern, microstructure

## Abstract

Fish collagen has been widely used in tissue engineering (TE) applications as an implant, which is generally transplanted into target tissue with stem cells for better regeneration ability. In this case, the success rate of this research depends on the fundamental components of fish collagen such as amino acid composition, structural and rheological properties. Therefore, researchers have been trying to find an innovative raw material from marine origins for tissue engineering applications. Based on this concept, collagens such as acid-soluble (ASC) and pepsin-soluble (PSC) were extracted from a new type of cartilaginous fish, the blacktip reef shark, for the first time, and were further investigated for physicochemical, protein pattern, microstructural and peptide mapping. The study results confirmed that the extracted collagens resemble the protein pattern of type-I collagen comprising the α_1_, α_2_, β and γ chains. The hydrophobic amino acids were dominant in both collagens with glycine and hydroxyproline as major amino acids. From the FTIR spectra, α helix (27.72 and 26.32%), β-sheet (22.24 and 23.35%), β-turn (21.34 and 22.08%), triple helix (14.11 and 14.13%) and random coil (14.59 and 14.12%) structures of ASC and PSC were confirmed, respectively. Collagens retained their triple helical and secondary structure well. Both collagens had maximum solubility at 3% NaCl and pH 4, and had absorbance maxima at 234 nm, respectively. The peptide mapping was almost similar for ASC and PSC at pH 2, generating peptides ranging from 15 to 200 kDa, with 23 kDa as a major peptide fragment. The microstructural analysis confirmed the homogenous fibrillar nature of collagens with more interconnected networks. Overall, the preset study concluded that collagen can be extracted more efficiently without disturbing the secondary structure by pepsin treatment. Therefore, the blacktip reef shark skin could serve as a potential source for collagen extraction for the pharmaceutical and biomedical applications.

## 1. Introduction

Marine organisms cover three-fourths of the land surface, claiming to be a treasure for many biologically active substances, especially proteins and carbohydrates. The extraction of proteins from marine sources has been increasing at a tremendous rate due to their distinct environment and biological activity. For instance, collagens from fish species have been explored by researchers for many years to sort-out the suitable materials for biomaterial fabrication in tissue engineering applications. It is well-known that collagen, the most abundant structural protein in the extracellular matrix (ECM), is the main component of various connective tissues in the body [1,2]. Based on the structure, molecular composition and distribution, collagens are classified according to at least 29 different types [3]. However, the main backbone of most of the collagens is composed of chains of polypeptides with the repeating sequence (Gly-X-Y)n, where X and Y are commonly occupied by proline and hydroxyproline [1]. Collagen from the skins of several fish species such as tilapia [4], horse mackerel, yellow sea bream, and tiger puffer [5], black ruff (*Centrolophus niger*) [6], and Parang-Parang (*Cirocentrus dorab*) [7] were reported earlier.

Much empirical evidence proved the adaptability of fish collagen for the fabrication of different biomaterials for artificial tissue implants. For instance, different types of scaffolds were fabricated using collagens from tilapia [8,9], *Trachicephalus uranoscopus* [10], bigeye snapper *Priacanthus hamrur* [11], etc. Fish collagen has been used as an alternative to mammalian counterparts for food, biomedical and pharmaceutical uses due to safety reasons and religious constraints [12]. In addition, fish processing industries produce large quantities of byproducts, in which most of the sources such as skin, bones, and scales are poorly utilized [13].

The blacktip reef shark (*Carcharhinus melanopterus*) belongs to the Carcharhinidae family, a species of requiem shark which is commonly found in Pacific regions like southern Asia, the Philippines and northern Australia. Shark is commonly used for shark fin and fillet production. During shark processing, the generated wastes, particularly skin and cartilage, can be ultimately used as potential source material for collagen extraction [14,15]. Unfortunately, none of the studies has used blacktip reef shark skin for collagen extraction and, therefore, it is important to understand the properties of collagens extracted from blacktip reef sharks, due to its potential value. Hence, this study is intended to accomplish the above objectives to extract the collagens using acetic acid and pepsin. It is important to investigate the physicochemical and functional properties of collagens that are extracted in two different ways: with and without enzymatic digestion. Therefore, in the present study, we tried to extract and characterize acid-soluble and pepsin soluble collagen from Blacktip reef shark (*Carcharhinus melanopterus*) skin, and provided a simultaneous comparison of collagens. Accordingly, the present results would be useful in developing a viable alternative collagen material for further biomedical and pharmaceutical applications.

## 2. Results

### 2.1. Protein Pattern of Collagens

**Figure 1:**
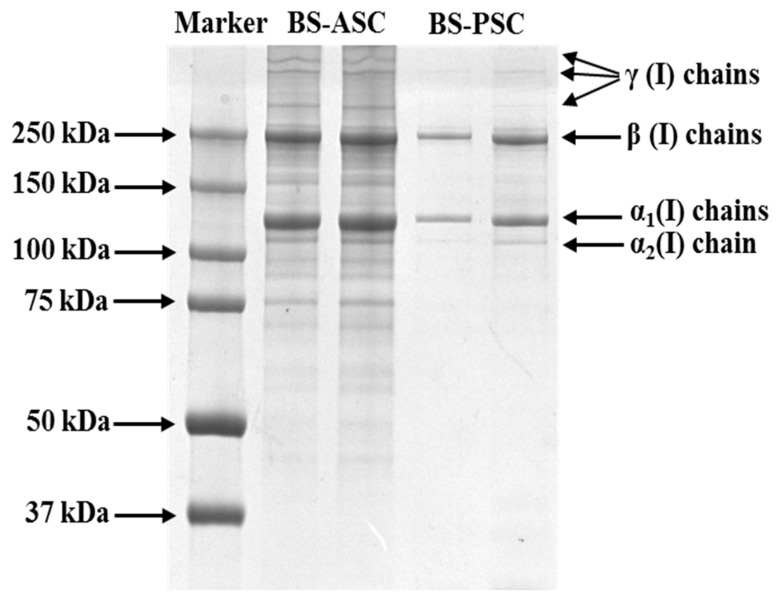
SDS-PAGE patterns of ASC and PSC from the skin of blacktip reef shark on 7.5% gel. Lane 1: protein markers; lane 2: BS-ASC (0.5 mg/mL); lane 3: BS-ASC (1 mg/mL); lane 4: BS-PSC (0.5 mg/mL); lane 5: BS-PSC (1 mg/mL).

The protein pattern between ASC and PSC was compared by using the SDS-PAGE method. ASC and PSC had similar molecular patterns, having α_1_, α_2_, β and γ chains, which confirm that both collagens belong to the type I category (Figure 1).

By comparing the standard molecular weight marker and standard analysis, the molecular weights of α1, α2, and β were about 135, 120 and 250 kDa, respectively. As expected, increasing collagen concentration from 0.5 mg/mL to 1 mg/mL had increased the bandwidth. Specifically, the pepsin treatment increased α and β band thickness compared to ASC, which claims the efficiency of pepsin in collagen extraction. As evidence, the final yield of PSC was higher (17.78% ± 0.64%) than ASC (15.46% ± 0.42%) (data are shown as mean ± standard deviation, *n* = 3, *p* < 0.05). However, the intact structure of collagen was more stable in ASC than in PSC, since the pepsin treatment had triggered the strong hydrolysis of collagen; as a result, many smaller peptide fractions were generated in PSC compared to ASC. From the gel image, it was clear that the higher molecular weight of collagen β and γ could be disintegrated into smaller fractions in PSC, which was not obvious in ASC.

### 2.2. Amino Acid Composition of Collagens

The total amino acid residues present in collagens were determined to understand the pattern of amino acid composition in ASC and PSC. In general, the pattern of the amino acid profile was similar between ASC and PSC. For instance, both collagens had a higher content of glycine as a major amino acid, only the percentage varies in between collagens, having higher content in PSC (293 residue/1000 residues) than ASC (283 residue/1000 residues) (Table 1). The second major amino acid was hydroxyproline (195 and 202 amino acid residue/1000 residues for ASC and PSC, respectively), followed by alanine, proline and glutamic acid, respectively.

There was no sulfur-containing amino acid observed in either collagen. From the results, it was clear that the hydrophobic amino acids such as glycine, proline, alanine, valine, leucine, isoleucine, and phenylalanine were more dominant than hydrophilic amino acids such as serine and threonine in both collagens. Compared to ASC, the content of amino acid was in general higher in PSC, which also supports the higher yield and liberation of peptide fragments in PSC (Figure 1). The content of imino acids such as proline and hydroxyproline in PSC (311.68 residues/1000 residues) was much higher than in ASC (288.14 residues/1000 residues).

### 2.3. Maximum Absorption of Collagens

**Figure 2:**
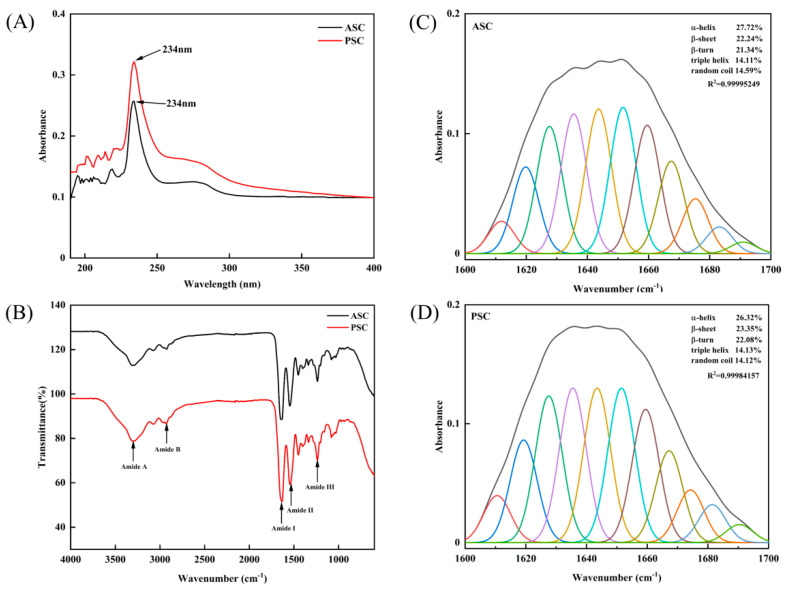
(**A**) UV–Vis spectrum of ASC and PSC made from the skin of the blacktip reef shark. (**B**) Fourier transform infrared spectra of BS-ASC and BS-PSC. Secondary structure analysis of ASC (**C**) and PSC (**D**) through the deconvolution of amide I band (between 1600 and 1700 cm^−1^).

This experiment was performed to investigate the two characteristic features of collagens: (1) to identify the maximum absorption (nm) of collagen and (2) to verify the contamination of non-collagenous protein presence in extracted collagens. The results showed that the collagens had maximum absorbance at 234 nm, respectively (Figure 2A). The absorption intensity was more in PSC at 230 nm than in ASC. There was no absorbance at 280 nm that usually corresponds to a sulfur-containing amino acid, cysteine, which is normally absent in collagen. From the above finding, it was further confirmed that the extracted collagen was pure.

### 2.4. Secondary Structure Analysis

The structural changes of collagens were investigated by using FTIR spectra. FTIR transmission spectra of ASC and PSC were shown in Figure 2B and Table 2. Both collagens had general transmission patterns in major amide bands such as amide A, amide B, amide I, amide II and amide III, respectively. The maximum transmission wave numbers of amide A and amide B (which represent N-H stretch and CH_2_ asymmetric stretch, respectively), were at 3298 and 2926 cm^−1^, and 3298 and 2930 cm^−1^ for ASC and PSC, respectively. ASC and PSC had maximum transmission for amide I at 1639 cm^−1^, amide II at 1542 and 1546 cm^−1^ and amide III at 1237 cm^−1^, respectively. There were not many differences observed in amide I, II and III bands between ASC and PSC. The IR absorption ratios of two collagens were 1.11 (ASC), and 1.03 (PSC), respectively.

In addition, the secondary structural pattern of collagen was determined by using PeakFit Version 4.12 software and the Gaussian peak fitting algorithm. The data showed that ASC and PSC contained 27.72 and 26.32% of α helix, 22.24 and 23.35% of β-sheet, 21.34 and 22.08% of β-turn, 14.11 and 14.13% of triple helix and 14.59 and 14.12% of the random coil, respectively (Figure 2C).

### 2.5. Solubility against pH and Salt

**Figure 3:**
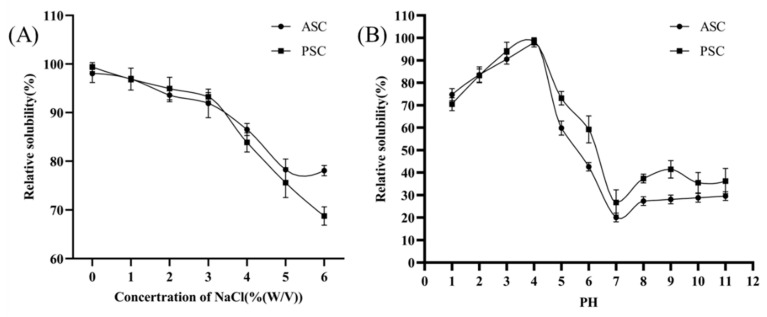
Relative solubility (%) at different pH values (**A**) and NaCl concentrations (**B**) of ASC and PSC isolated from blacktip reef shark skin extracted using different methods.

The functional behavior of collagen is generally investigated by determining pH and salt solubilities. For this intention, collagen was solubilized with different salt concentrations (ranging from 0–6%) and pH (ranging from 1 to 11), respectively. In general, increasing salt concentration from 0–6% decreased the solubility of collagen from 100% to 70% (Figure 3A). Both collagens reach the exponential phase (optimum) at 3% NaCl; after that, a sudden decrease in the solubility of collagens was observed. On the other hand, a typical sigmoid curve was observed in a collagen solubility pattern against pH (Figure 3B). Similar to salt, both collagens had no significant changes in solubility against pH, and the maximum solubility was obtained at pH 4.

### 2.6. Peptide Mapping

**Figure 4:**
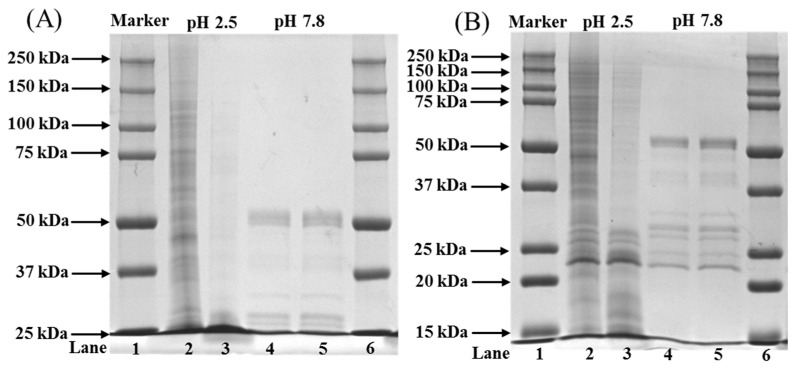
Peptide maps of ASC and PSC from the skin of blacktip reef shark digested by trypsin using 7.5% (**A**) and 12% (**B**) gels. Lanes 1 and 6: protein markers; lanes 2 and 4: ASC; lanes 3 and 5: PSC. Lanes 2 and 3: peptide fragments of collagens with trypsin digestion at pH 2.5; lanes 4 and 5: peptide fragments of collagens with trypsin digestion at pH 7.8.

Peptide mapping was used to understand the hydrolysis pattern of collagen by proteolytic enzymes. Based on the earlier protocol, the collagen was hydrolyzed by trypsin with two different pHs at 2.5 and 7.8 for 3 h and 3 min. Due to the higher activity of trypsin at neutral or slightly basic pH (6–7.5), the reaction was carried-out at pH 7.8 for a shorter time. As shown in Figure 4A, the peptide map was not so obvious in all the collagens hydrolyzed by trypsin at pH 2.5 and 7.8, except ASC at pH 2.5. The hydrolytic pattern of ASC was significantly different from PSC, which had no peptide bands, unlike ASC (Figure 4A). The unseen peptide bands of collagens were visible as the gel concentration increased from 7.5 to 12% (Figure 4B). The data showed that proteolytic hydrolysis of ASC released the low MW peptide fragments ranging from 200 to 5 kDa MW at 2.5 pH, whereas the peptide fragments from 55 to 23 kDa MW were observed at pH 7.8 for ASC (Figure 4B). In general, all the collagens had a major peptide fragment at 23 kDa, both collagens had similar peptide fragments ranging from 55 to 23 kDa at pH 7.8, and no higher molecular component was seen in any of the groups.

### 2.7. Microstructural Analysis

**Figure 5:**
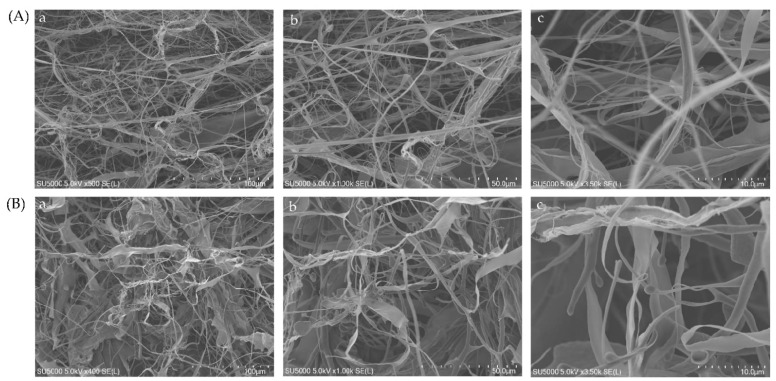
Scanning electron microscopic structure of ASC (**A**) and PSC (**B**) from blacktip reef shark skin. SEM image with different magnifications: (**a**) (100 μm), (**b**) (50 μm), (**c**) (10 μm).

**Figure 6:**
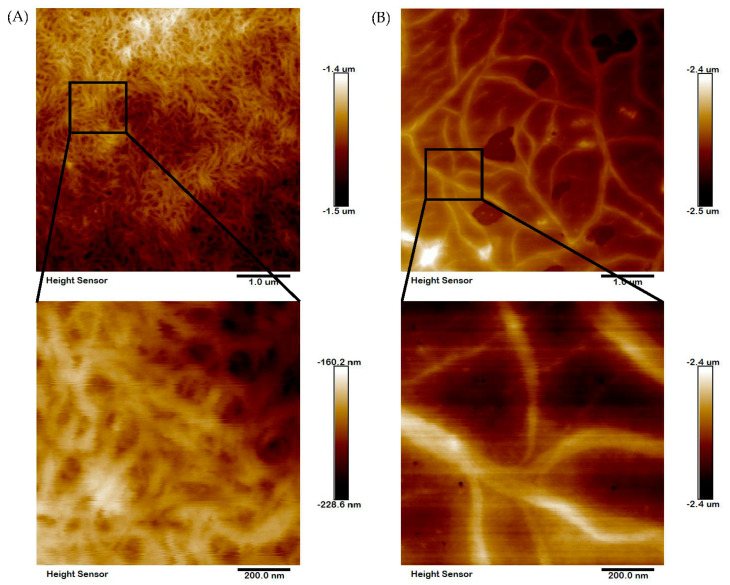
High-resolution AFM image of blacktip reef shark skin collagen fiber bundle. The ASC (**A**) and PSC (**B**) fibril display the natural structure.

The microstructural features of collagens were determined by using SEM at different magnifications (100, 50 and 10 μm). It was seen that both collagens had a fibrillary and more condensed network-like structure (Figure 5). The distribution of fiber was more homogeneous and alveolate porous. There were more interconnected filaments with varying thicknesses in both collagens. As seen, there were not many changes in the microstructural characteristics of either collagen. The fibril bundle structure of collagen was further confirmed by the AFM experiment. Similar to SEM, the fibril bundle structure was denser and more condensed in nature in both ASC and PSC (Figure 6). However, the AFM image of PSC showed longer filaments with loosely connected intra-structure than ASC, where the filaments were shorter and more connected to each other.

## 3. Discussion

From the protein pattern, we confirm that the extracted collagens were characterized as type-I collagen due to the presence of α1, α2, β and γ chains. To support this finding, earlier reports dealing with fish skin type-I collagens had a similar molecular pattern of proteins [20,21,22]. The SDS-PAGE protein profile further confirmed that the extracted collagen was pure and had no presence of other proteins. The pattern of the amino acid composition of blacktip reef shark skin collagen was very similar to the earlier reported fish collagens [22]. The higher amount of amino acid content in PSC was related to the extensive hydrolysis process of raw materials by pepsin, which ultimately facilitated the solubilization process of collagen in raw material and thereby releases many smaller fractions.

The maximum absorbance of collagen at 234 nm was due to higher amounts of glycine (which absorbs maxima at 216.8 nm) and peptide bonds (at 230 nm) [23]. In addition, the liberation of more peptides (which tend to absorb UV maxima at 230 nm) by pepsin treatment might be the actual reason for the higher absorption of PSC at 234 nm than ASC. Interestingly, no absorbance peak of collagens at 280 nm further confirmed that the extracted collagens were pure.

FTIR analysis confirmed that the secondary structure of collagen was not significantly altered by the different extraction procedures with acid and pepsin. Only CH_2_ asymmetric stretch and N-H bend coupled with C-N stretch were more pronounced in PSC than ASC, which was due to the extensive hydrolysis of collagen by pepsin. The IR absorption ratio indicated that the triple helix and high molecular structure of the two collagens were intact [24]. The amide I band in the wavelength range from 1600 to 1700 cm^−1^ was used to calculate the collagen secondary structure [25,26]. Using the Gaussian peak fitting algorithm, we confirmed that neither collagen had any significant changes in α helix, β-sheet, β-turn, triple helix and random coil, which confirms that the pepsin treatment had not significantly altered the secondary and triple helical structure of collagen and only improved the production yield. A recent study also reported a similar finding in the secondary structures of α helix, β-sheet, β-turn and triple helix by pepsin soluble collagen [27]. This finding reveals the compatibility of pepsin use in collagen extraction from blacktip reef shark skin.

Increasing ion concentration on the surface reduces the free functional groups and the hydrophilic nature, thereby reducing the solubility of collagens [28]. The maximum solubility of collagen against pH was observed at 4, which corresponded to the isoionic point of collagens; for instance, it was reported that the isoionic point of collagen ranged from pH 3 to 5 [29,30].

In the present study, the collagens were hydrolyzed at pH 7.8 by trypsin for a shorter duration (3 min) unlike pH 2.5 (3 h), due to the higher proteolytic activity of trypsin at pH 7.8 [31]. The peptide fragments observed in the present study were similar to the peptide maps generated in earlier studies [32,33]. At pH 2.5, the peptide fragments were derived from higher MW components; as evidence, the intensity of high MW bands such as α, β and γ was decreased significantly and almost absent in collagens treated at pH 7.8. To support this finding, earlier studies reported similar findings with the absence of a higher MW component after trypsin hydrolysis [34,35,36]. The lower amount of peptide bands observed in PSC at pH 2.5 compared to ASC was mainly attributed to the earlier hydrolysis of collagen by pepsin during extraction, which makes them more susceptible to consecutive hydrolysis by another proteolytic enzyme, trypsin, to liberate peptides less than the measurable range (below 15 kDa). In contrast, the lower amount of peptide fragments in collagens treated at pH 7.8 than at pH 2.5 were due to the extensive hydrolysis of collagen by trypsin at an optimum pH of 7.5 and thereby smaller peptides could be obtained (less than 15 kDa). To support this finding, earlier studies reported a similar observation of peptide mapping of collagens (ASC and PSC) extracted from fresh Spanish mackerel [31], and largefin longbarbel catfish [37]. The similar microstructural properties of ASC and PSC proved that the pepsin treatment did not contribute to any major changes in the microstructure, as evidenced by FTIR data earlier (Figure 3).

## 4. Materials and Methods

### 4.1. Chemicals

Sodium hydroxide (NaOH), acetic acid, sodium chloride (NaCl), hydrochloric acid (HCl), ethanol, and potassium bromide (KBr) were purchased from Sinopharm Chemical Reagent Co., Ltd. (Shanghai, China). Pepsin from porcine stomach mucosa (EC 3.4.23.1; 1:3000 U) was purchased from Beijing Solarbio Science & Technology Co., Ltd. (Beijing, China). Trypsin (EC 3.4.21.4, 1:250), dithiothreitol (DTT), and Coomassie brilliant blue R-250 were purchased from Sigma-Aldrich Corporation (St. Louis, MO, USA). Dual Color protein standard marker with MW of 37–250 kDa (Catalog No. 1610374), 4× Laemmli Sample Buffer (Catalog No. 1610747), 10× Tris/Glycine/SDS (Catalog No. 1610732) were purchased from Bio-Rad Laboratories Inc. (Hercules, CA, USA). All reagents were used at an analytical grade unless otherwise specified.

### 4.2. Raw Materials and Pre-Treatment

The skin of the blacktip reef shark (*Carcharhinus melanopterus*) was purchased from M/s. Yueqing Ocean Biological Health Care Product Co., Ltd. Zhejiang, China. The skins were washed thoroughly with tap-water and cut into small pieces, then stored in plastic bags in the freezer at −80 °C until further use. The skin pieces of blacktip reef shark were mixed with 0.1 M NaOH at a sample-to-solution ratio of 1:10 (*w*/*v*) at 4 °C with stirring for 24 h to remove water-soluble substances and non-collagenous protein, respectively, and the alkali solution was refreshed every 8 h. The samples were then repeatedly washed with distilled water until a neutral pH of washing water was obtained.

### 4.3. Preparation of Acid-Soluble and Pepsin-Soluble Collagen

All procedures were carried out at 4 ℃ with stirring. Acid-soluble (ASC) and pepsin-soluble collagens (PSC) were extracted from blacktip reef shark skin according to our earlier method with some modifications [38]. For extraction of acid-soluble collagen, the cleaned skins were soaked with 0.5 M acetic acid in a ratio of 1:20 (*w*/*v*) for 48 h. The mixture was then centrifuged at 20,000× *g* for 30 min at 4 °C using a Himac CR 21G High-speed floor centrifuge (Hitachi, Tokyo, Japan).

**Figure 7:**
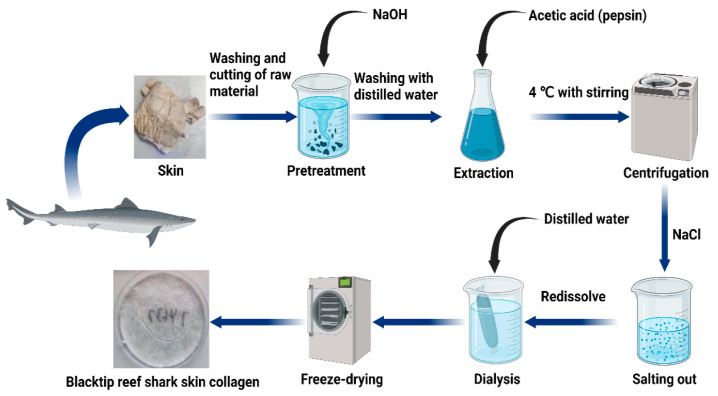
Schematic representation of steps involved in collagen extraction.

At the same time, the centrifugation pellet from the first extraction was redissolved in 0.5 M acetic acid for second extraction for 48 h, and then the supernatant was combined with the earlier extraction. After centrifuge, the collagen was precipitated by mixing the supernatant with 1.0 M NaCl. The salting-out precipitates were redissolved in 10 volumes of 0.5 M acetic acid and dialyzed using dialysis membranes (MWCO: 10 kDa) against distilled water until a neutral pH was obtained. The dialyzed sample was lyophilized using a freeze-dryer (Labconco Freezone 2.5L, Kansas City, MO, USA), and concentrates were stored at −80 °C. The procedure for the extraction of pepsin soluble collagen was the same as the extraction of acid-soluble collagen by using 1% pepsin in 0.5 M of acetic acid. A detailed flow chart of the blacktip reef shark skin collagen preparation procedure is presented in Figure 7.

### 4.4. Molecular Pattern

The molecular pattern of purified collagen was determined by using sodium dodecyl sulfate-polyacrylamide gel electrophoresis (SDS-PAGE) according to Laemmli’s method [39]. The ASC and PSC samples were prepared with SDS to obtain 0.5 mg/mL and 1 mg/mL, respectively. The collagen samples were mixed with (3:1) 4 × Laemmli Sample Buffer (SDS, Tris-HCl, bromophenol blue, glycerol, and DTT) and oscillated slightly with a scroll oscillator (TianGen Biotech Co, Ltd., Beijing, China). The samples were boiled for 5 min and the boiled mixture was briefly centrifuged at 1890 g. The test samples and standard protein standard marker (MW ranging from 37 kDa to 250 kDa) (Bio-Rad Laboratories Inc., Hercules, CA, USA) were loaded onto 4.5% stacking polyacrylamide gel with 7.5% separating gel (Cat# PG112, EpiZyme Biotechnology, Shanghai, China). The electrophoresis unit was set at a constant voltage of 200V in order to efficiently separate the collagen samples using a Mini-PROTEAN Tetra Cell (Bio-Rad Laboratories Inc., Richmond, CA, USA). After the electrophoresis, the gel was stained with 0.25% Coomassie brilliant blue R-250 solution for 30 min and discolored with the mixture of 20% (*v*/*v*) ethanol and 10% (*v*/*v*) acetic acid twice, each for 1 h until clear protein bands were observed. The protein bands were then captured using the gel documentation system (Clinx GenoSens 2100(T), Shanghai, China).

### 4.5. Amino Acid Composition

The amino acid content of collagen samples was analyzed by using an amino acid analyzer (Hitachi LA-8080, Tokyo, Japan) [14]. The freeze-dried collagens were completely hydrolyzed in 6M HCl at 110 °C for 24 h. The excess amount of solvent was evaporated under the vacuum incubator until the dried sample then dissolved in distilled water and the drying process was repeated three to four times. In the end, the dried sample was dissolved with a minimum amount of sodium citrate buffer solution (pH 2.2) and filtered through a 0.45 nm hydrophilic membrane. The amino acid analyzer was calibrated with a standard reagent, and a positive control of all amino acids was run as a reference before analyzing the test sample. The retention time of each amino acid peak was equalized with the respective positive control amino acid peak and the content of amino acid is expressed as the number of residues/1000 residues.

### 4.6. UV Absorption

The maximum absorption of collagen in the UV range was determined using a UV-vis spectrophotometer in order to confirm the purity and contamination of other proteins [33]. The ultraviolet absorption spectra of blacktip reef shark skin collagens were recorded by a spectrophotometer (MAPADA UV-3000PC, Shanghai, China). Freeze-dried collagen was dissolved in 0.5 M acetic acid and the absorption from 190 to 400 nm was measured at a scan speed of 2 nm/s with an interval of 1 nm. An aliquot of 0.5 M acetic acid was used as blank.

### 4.7. Fourier Transform Infrared Spectroscopy (FTIR)

The structural and amide differences between ASC and PSC were determined by using the Spotlight 400 FT-IR Imaging System (PerkinElmer, Waltham, MA, USA) equipped with a deuterated triglycine sulfate detector. The 2 mg freeze-dried sample was mixed with dried KBr (100 mg) in order to make a 13 × 1 mm thin transparent disk by subjecting a pressure of approximately 5 × 10^6^ Pa. The transparent disk was then placed in a sample holder in FTIR and the spectra in a range of 4000 to 600 cm^−1^, with automatic signal gain collected in 32 scans at a resolution of 2 cm^−1^. The absorption intensity of the peaks was calculated using the baseline method. The resultant spectra were analyzed using Origin Pro 2021 software. The secondary structures of the collagen were analyzed through the areas of 1600–1700 cm^−1^ in the amide I region using PeakFit Version 4.12 software (SeaSolve software Inc., Framingham, USA) and the Gaussian peak fitting algorithm. Finally, the secondary structure percentage was calculated by dividing the peak area of the secondary structure by the whole peak area of all the secondary structures.

### 4.8. Relative Solubility

The relative solubility of collagen was determined by varying NaCl and pH by following our previous protocol [40]. The freeze-dried collagen sample (10 mg/mL) was prepared with 0.5 M acetic acid with gentle stirring at 4 °C for 12 h and used for solubility experiments.

#### 4.8.1. Effect of pH

The collagen solution (5 mL) was transferred into a series of centrifuge tubes, adjusted to pH values ranging from 1 to 11 by the addition of the appropriate amount of 6 M NaOH or 6 M HCl. The resulting sample solution totaled 10 mL of distilled water. The solution was stirred gently for 30 min at 4 °C and centrifuged at 5000× *g* for 30 min. An aliquot (1 mL) of the supernatant was collected from each tube and the protein content was measured. The relative solubility of collagen was calculated compared with the pH rendering the highest solubility.

#### 4.8.2. Effect of NaCl

The collagen solution (5 mL) was mixed with 5 mL of cold NaCl in acetic acid of various concentrations (0–12%, *w*/*v*) to obtain final concentrations of 1–6% (*w*/*v*). The mixture was stirred gently at 4 °C for 30 min and centrifuged at 10,000× *g* for 30 min at 4 °C. The relative solubility was calculated compared with that of the salt concentration exhibiting the highest solubility.

### 4.9. Peptide Mapping

The peptide mapping pattern of collagen against the proteolytic enzyme, trypsin, was determined by following the method of Li et al., with some modification [31]. The freeze-dried collagen sample was dissolved in 0.5 M acetic acid, pH 2.5, at a concentration of 1.5 mg/mL. In parallel, another set of collagen samples was dissolved in a 0.2 M sodium phosphate buffer, pH 7.8, at a concentration of 1.5 mg/mL. After the addition of trypsin with an enzyme/substrate ratio of 1/2 (*w*/*w*) to collagen solutions, the reaction mixtures were incubated at 37 °C for 3 h (pH 2.5) and 3 min (pH 7.8), respectively. The SDS-PAGE sample buffer was added to the digestion samples, and the mixtures were boiled for 5 min to terminate the reaction. Using 4.5% stacking gel, 7.5% and 12.5% separating gels, SDS-PAGE was performed to separate peptides generated by the protease digestion and compared.

### 4.10. Microstructural Analysis

The changes in the structural properties between ASC and PSC were determined by scanning electron microscopy (SEM). The freeze-dried collagen samples were pasted on an aluminum plate with conductive adhesive tape in the desired orientation and placed in an ion coater for the gold coating to increase the electrical conductivity. The metalized collagen samples on the SEM sample holder (20-s glow discharged carbon support adhesive films) were mounted and then introduced into the specimen chamber for analysis. The surface morphology of collagen was captured with different magnifications of 100 μm, 50 μm and 10μm by using SEM (Hitachi SU5000, Tokyo, Japan) at an accelerating voltage of 15 kV.

### 4.11. Atomic Force Microscope Analysis

In order to evaluate the surface morphology, collagen fiber alignment, topography and superficial properties of the collagen, an atomic force microscope (AFM, Bruker Corporation, Billerica, MA, USA) was carried-out with collagen samples by following the standard operating procedure [41]. SNL-series silicon 1 cantilevers with a normal spring cantilever of 0.35 N/m were used. The nominal AFM tip radius is 2 nm and the maximized tip radius is 12 nm. 10 μL collagen aqueous solution was cast on freshly cleaved muscovite mica and was allowed to dry in ambient air for 60 min. Images were obtained using a multimode scanning probe microscope with NanoScope Analysis software (version 1.80, Bruker Corporation, Billerica, MA, USA) operating in the tapping mode, in air, at room temperature. Surface images were acquired at fixed resolution (512 × 512 data points) with a scan rate of 1.0 Hz.

### 4.12. Statistical Analysis

Each experiment was replicated three times. Data were expressed as mean ± standard deviation and mentioned in the figure legends. Statistical analysis was performed using GraphPad Prism 9 software (GraphPad Inc, San Diego, CA, USA) using two-way ANOVA with Fisher’s LSD test multiple comparison analysis. The values identified as outliers were excluded from statistical analysis. Results were considered statistically significant if the *p*-value < 0.05.

## 5. Conclusions

In this study, the structural and functional changes in ASC and PSC were investigated, and it was found that the use of pepsin had contributed to the increase in the collagen yield; however, it produced many smaller protein fragments after extraction. Therefore, the functional and structural properties were investigated in ASC and PSC, and no significant differences were observed in both collagens, which directly revealed that the pepsin treatment did not affect the secondary structures such as α helix, β-sheet, β-turn, and triple helix. Therefore, the physicochemical, structural and functional properties of collagens indicate that the PSC could be an appropriate material for biomaterial fabrication in tissue regeneration applications.

## Figures and Tables

**Table 1:** Amino acid composition of acid-soluble and pepsin soluble collagens from blacktip reef shark skin (residues/1000 residues).

Amino Acid	Acid Soluble Collagen	Pepsin Soluble Collagen
Leucine (Leu)	23.24 ± 0.13 ^a^	20.52 ± 0.41 ^b^
Isoleucine (Ile)	18.05 ± 0.70 ^a^	17.24 ± 0.01 ^b^
Phenylalanine (Phe)	11.07 ± 0.34 ^a^	10.73 ± 0.16 ^a^
Valine (Val)	21.53 ± 1.04 ^a^	19.45 ± 0.09 ^b^
Methionine (Met)	3.37 ± 0.16 ^a^	3.21 ± 0.15 ^a^
Tyrosine (Tyr)	1.69 ± 0.32 ^a^	1.06 ± 0.14 ^b^
Alanine (Ala)	103.94 ± 0.07 ^a^	105.04 ± 0.05 ^b^
Threonine (Thr)	20.46 ± 0.53 ^a^	19.91 ± 0.27 ^b^
Glutamic acid (Glu)	66.63 ± 0.29 ^a^	64.74 ± 0.06 ^b^
Glycine (Gly)	292.95 ± 0.46 ^a^	283.86 ± 0.19 ^b^
Serine (Ser)	38.49 ± 0.15 ^a^	36.22 ± 0.15 ^b^
Aspartic acid (Asp)	38.30 ± 0.28 ^a^	36.43 ± 0.10 ^b^
Arginine (Arg)	43.86 ± 0.2 ^a^	43.00 ± 0.15 ^b^
Lysine (Lys)	21.13 ± 0.54 ^a^	20.41 ± 0.34 ^b^
Histidine (His)	7.14 ± 0.32 ^a^	6.50 ± 0.20 ^b^
Proline (Pro)	195.84 ± 0.47 ^a^	202.22 ± 0.37 ^b^
Hydroxyproline (Hyp)	92.3 ± 0.16 ^a^	109.46 ± 0.13 ^b^
Total	1000.00	1000.00
Imino acid	288.14 ± 0.31 ^a^	311.68 ± 0.24 ^b^

All values are shown as mean ± standard deviation (*n* = 3, ^a^ and ^b^ in the same row indicate significant differences, *p* < 0.05).

**Table 2:** FTIR spectra peak position and assignments for blacktip reef shark acid-soluble collagen (ASC) and pepsin-soluble collagen (PSC).

Region	Wavenumber (cm^−1^)		Assignment	References
	ASC	PSC		
Amide A	3298	3298	N-H stretch	Doyle et al. [16]
Amide B	2926	2930	CH_2_ asymmetrical stretch	Abe and Krimm [17]
Amide I	1639	1639	C=O stretch	Muyonga et al. [18]
Amide II	1542	1546	N-H bend coupled with C-N stretch	Jackson et al. [19]
Amide III	1237	1237	N-H in-plane bend	Jackson et al. [19]

## Data Availability

Not applicable.
